# The Condition of Sleeplessness and Its Treatment

**Published:** 1906-07

**Authors:** Woodbridge Hall Birchmore

**Affiliations:** Brooklyn, N. Y.


### The Condition of Sleeplessness and Its Treatment.

### A Note in Preliminary.

BY WOODBRIDGE HALL BIRCHMORE M. D., Brooklyn, N. Y.

PERHAPS there is no physical state of the human body less understood than sleep, and beyond the fact that it is in some way connected with the nutrition of the group of organs which we call nervous, the nervous system, we know very little about it. In the middle of the last century, sleep seems to have been a favorite subject for speculation to a number of physicians, the members of a small group of writers, but it does not appear from their writings that these men who wrote so freely on this subject were adding anything of importance to the common fund of ascertained facts.

Sleep appears to be a condition of the organism during which many functions go on in regular fashion. I have seen a man sleep soundly while playing waltz music on the piano for dancers; this nap did not end until the end of the set of waltzes, and then he wakened with a start. The fact that one can sleep on horse-back is within the experience of most of us.

The condition of the somnambule is a proof that sleep does not suppress co-ordination in muscular actions, possibly not even the centripetal path of special sense, and it is within the bounds of observed fact to declare that there is no inherent absurdity in the often repeated statement that the somnambule would not have risked while waking that which he (or she) did safely while asleep.

Dr. Phelps's contention that the prefrontal lobe on the left is the physical location of those nervous actions which we commonly describe as mental if absolutely correct gives us a distinct foundation for a logical theory of sleep, as soon as the distinction between "mental processes" and consciousness shall be ascertained, because some mental processes seem to be quite independent of consciousness and hardly voluntary, and these do surely continue during sleep; and who has not gone to sleep thinking upon the possible solution of some problem to find the complete solution his first waking thought?

It has been suggested by some well known writers that sleep represents the nutrition period of the brain; perhaps of the whole body, but of the brain in particular, during which period the cells were reconstructing the cell-contents or that part of it, the "annexed molecules", the fuel (sic), which has been exhausted by their special functional activity.

Interesting as such speculations are they are speculations only, nothing more they lead not whither, yet the phenomenon which we call sleep certainly represents a state, action or condition essential to the maintaining of the functional integrity of the body, (and the form of functional activity which we call mind, as a bodily activity), that is maintaining the nutrition processes in the condition which we call health.

Sleeplesness is for the writer the absence of this condition, it is more or less distinctly the expression of disordered nutrition. Wakefulness is physiological, sleeplessness is pathological, it is only in the dictionary that they are synonyms.

In the artificial conditions under which we live those nutrition processes which in the hypothetical state of nature must have had the first consideration are crowded to one side. The savage does not dine, he feeds and feeds until he can feed no more; and then he sleeps, and sleeps until his food has been digested and even assimilated; and then he is ready for renewed and violent exertion. The ill effects produced upon those habituated to "dinner at six" by a heavy meal at mid-day, which is often followed by an overpowering demand for a chance to sleep, are really due to the changed and unaccustomed environment of the nerve cells, which are now irrigated with a blood rich in food, vice that to which at this time of the day they find themselves wonted. But the artificial environment requires that the "whole administration shall live up to the time card," the wants of the body, its nutrition conditions must be met, and met on time. It is this fact which makes sleeplessness so serious a matter in the terribly fast paced life of today.

It follows that to meet the demands of modern life and its methods, a life whose every act is paced by the automobile and regulated by watches so accurate that those which we buy for two dollars are made to run within the limits set for a ship's chronometer a century and a half ago, sleep also must come on time and go. This is the case because our bodies to a large extent must be adapted to the laws which regulate our minds' activities, and if these our bodies are found wanting in this adaptability we suffer, often to a degree which those more fortunate fail totally to understand.

Sleeplessness may take the form of delayed somnolency, or the unfortunate will waken after a period of sound slumber, and after remaining awake for an indefinite period will again fall asleep, to waken weary and unrefreshed. The complaint appears absurd to one who cannot by his own experience appreciate the full value of the threatened injury. As I once heard a physician say "I wish you could have been in my room this morning. I had a patient complaining that she dreaded her bed for fear of not going to sleep at once." I hear the same complaint three or four times each week and it usually is a very serious, almost tragic, matter to the patient. I do wish I could get at the bottom of this trouble, a man who would make a specialty of treating sleeplessness, and who really could do it right, should make a fortune, but he would need to know how to do it—don't you know? There is no doubt of the reality of the trouble so far as I can see, and the misery of the unfortunates is real, and the suffering absolutely pitiful."

The more common and less serious form takes the shape of the complaint of inability to woo the drowsy god to the patient's bed. The patient is very much in earnest, "I assure you, Doctor, that there is nothing in my power to do to break up this trouble, which I am not ready to attempt. I used to go to sleep the instant my head touched my pillow, but now,—why Doctor, the other night I went to bed at eleven o'clock and a few minutes, but I was not yet asleep when the clock struck two." Or the complaint may be "I lay there in just a doze, thinking of every sort of horrible thing you can imagine." Although related, these are very distinct forms of sleeplessness and I have never known them to occur in the same person. In the first form the person is really awake; in the second all the faculties of sense are sleeping and this modification is closely allied to dreaming and nightmare; in some cases this semisleep, if such a phrase may be used, will not hinder bodily rest although the patient will waken in the morning "mentally worn out", to use their own phrase.

The second form of the trouble in which the night begins auspiciously, but deceitfully, is much the more serious and may be the prodrome of dangerous disorder; in fact it is a symptom whose warning should be most heedfully considered, for it is the proof of serious disorder in the nervous system's nutrition. There is a peculiar similarity in the accounts given by these unfortunates and having heard the tale of one, the physician can anticipate the narrative of all the rest. The patients all say the same thing as to the effect of an attack, "I did go to sleep finally, but when the time came to be awakened I was more weary than when I retired, in fact I was exhausted. Somehow I feel if this sort of thing continues I shall be seriously ill; usually I can drink my coffee without using my saucer, but when I have been awakened my hand trembles so that I cannot hold the cup steady, and when I wished to go to my tub I was so weak and undone that

I had to take hold of a chair to steady myself before crossing the room to the bath." There is no need of going further into details, every practitioner of experience knows the symptom picture, but there is one fact not so widely considered as it might be, that in the last group of cases the patient is suffering from acute fever of some sort, the temperature sometimes reaching 38.5 degrees C. (101.3 degrees F.) or even a degree higher.

It is equally worthy of notice that such an attack is terminated in some instances by the simple act of rising from bed, obtaining a glass of water, and drinking it. One lady who complained most bitterly of this condition of sleeplessness, best described as "vigillation," found complete relief in a large glass of "carbonic acid water." Another who had promised to call the doctor if she had an attack "to-night," rang the bell for the boy to send him for the doctor, then roused her maid and returned to her bed. When the doctor arrived the maid assured him that her mistress had "fallen asleep as soon as her head rested on the pillow." When in the morning the would-be patient, remembering that she had summoned the doctor yet wanting any recollection of his coming, asked her maid about the matter, she learned that the physician had answered the call at once, "so quickly that he must have been expecting that you would send for him, but you were sleeping soundly when he arrived, and he was much amazed thereby."

One patient, who was under my care for nearly three years and for various reasons, began to develop this habit of vigillation, greatly to her discomfort and it caused me much anxiety, but having taken a Seidlitz powder for another purpose she found that it relieved her vigil, and at the next attack of vigillation she made use of the same remedy with the same success. This may have been in some measure due to the therapeutic effect of the Seidlitz powder, or simply to the drinking of the cold water. Another patient in whom an attack of vigillation was invariably followed by nervous exhaustion, and headache, found in Abbott's Saline Laxative the same relief as did this one in the Seidlitz powder, and I have seen relief follow a glass of water, as was just above stated.

If we apply the word "sleeplessness" to the form of this trouble which prevents sleep and "vigillation" to that which breaks up and interferes with sleep, we have the needed distinctions in nomenclature, distinctions needed for intelligent discussion.

*The Treatment of Sleeplessness.*—To attempt the treatment of any condition intelligently we must have some notion of the cause, and it is my firm belief that the most frequent cause of sleeplessness is exhaustion, and the second hunger. It needs little more than observation, but little consideration of the facts observed to convince one that the condition of sleep follows naturally and logically the act of eating, the sequence being all but invariable in brutes, savages and children.

In regard to the effect of exhaustion, the over wearied doze, they do not fall asleep until somewhat rested, even if they have been fed, but dozing is just the act to which these patients (impatients, usually) object. They wish to fall into the arms of somnus the very instant after the desire becomes an act feasible. To them it is well to give a small dose of atropia sulphate, an eighth to half a milligram is usually quite enough. All that is desired is to give, for a few minutes, full relief to the nervous tension which prevents the circulation from establishing an equilibrium of the blood supply to the various organs involved. Without the atropia sulphate it may be an hour before the circulation takes on the rhythm of sleep, but with it sleep may come in a minute so to say. The very power of atropia makes it at times less useful than hyoscyamin: give this in a dose one-fourth to one milligram, permitting the pill or pellet to dissolve in the mucus-membrane-fold between the lower lip and the teeth. This fold is wonderfully supplied with absorbents and the drug is taken up about as fast as it goes into solution.

The relief to this sensation of nervousness, of irritability, it is this rather than of irritation, is the one demand of the patient, and if it can by chance be met by some means other than a narcotic it is of course wise to do so, and in certain cases it can be so met.

Experience shows that a small piece of sponge cake and a cup of coffee given to one in this state of irritation will in many cases act like a charm in sending them to sleep. Truly it would be simply uncalled for fatuity to say that the amount of food had been the talisman, it is the sensation of being fed which has given the relief. Does this give any clue to the method of action in the premises? We know that when food is taken into the stomach an immediate hyperemia of the whole digestive apparatus results. The amount of blood in the body being fixed, the amount that can be sent to the brain must be proportionally lessened, and this possibly aids in establishing that equilibrium which must be the natural law.

True we do not certainly know that the amount of blood distributed to the different brain organs is definitely diminished, but most certainly this is a very fair inference and if the blood supply is lessened the activity of the brain organs must be diminished also. But setting all speculation on the method of action aside, and frankly admitting that at the present state of our knowledge or our ignorance, any attempt at finality is impossible, the fact still remains that a small ration has relieved the nervous tension. Therefore I would suggest that, in this form of sleeplessness in which the patient becomes more and more irritable, and is so intensely nervous as to be at once a case of expectant attention and to really dread her couch lest she remain awake, I repeat, under these conditions, before drugs are used it is the part of wisdom to try feeding; if the first attempt does not succeed make another.

There is some reason for believing that the reason men are so seldom troubled in this way, i. e. with sleeplessness, is because men have no scruples in the matter of late suppers. If a man is convinced that his deslre at the moment is for a sandwich and a cup of coffee he sets about getting the said sandwich and cup of coffee and no Mrs. Grundy makes him afraid, yet I have heard a woman say "Have cake and coffee sent to my room at bedtime! at midnight! what will the others say?" My reply was, "Madame, your husband has his sandwich down stairs at fifteen minutes after midnight, why should you not have yours upstairs earlier?" Of course, such objections are simply formal.

A most convincing proof of the probability of this view was given me a few years ago while a guest in a large country house. One of the ladies, guests, sent me a note by her maid saying that she wished to see me professionally in the library, and of course, I went. She told a pitiful tale of sleeplessness, she was in the habit of taking a dose of chloral to secure sleep, and her immediate supply was exhausted. Reduced to plain words she was approaching the place, if she had not arrived, at which she would be dependent upon chloral for sleep. She said she realised the peril of her position but it was a case of no chloral, no sleep. I could see nothing in her physical condition to justify such a course and suggested that just for that night she should omit the chloral and make use of the sponge cake and coffee. At first she objected, saying that it would make talk if she asked for a lunch at such an unseasonable hour. I said,, "We men have ours, or something less innocent, downstairs at midnight. Just send your maid to the butlers' pantry and you will get what you want and no questions asked." She did as I suggested and during the morning she found means to assure me that she "had not had such a delicious sleep in months," and before dinner, 8 P. M., I had given the same advice to four other ladies one of them unmarried and just more than twenty, who assured me that she began using the chloral on a physician's advice, and another, a wife, about twenty-seven who assured me she had been using the chloral off and on for nearly four years, and that she did so without the knowledge of anyone but her maid. This case did not look at all promising but before my visit was ended I had the pleasure of knowing, if

I was justified in taking her word for it, that a cup of coffee mostly rich cream and a piece of cake or a large biscuit (Uneeda like but round), had been substituted for the dose of chloral, and that she had now such sleep as the chloral never gave. This group of cases was distinctly satisfying, because the men who were lunching just before going to bed showed no neurotic tendency but far otherwise, while this group of women were all of them beginning to show touches.

So much for the cases of true sleeplessness, these poor women were simply hungry; had they been of a social circle which rushes "the growler" they probably would never have needed the aid of chloral-hydrate, nor have heard of it. From my experience I say, "Try feeding first, then atropine, hyoscine, hyoscyamine, narceine, codeine, camphor-monobromide and cannabin in the order given."

*The Treatment of Vigillation.*—The treatment of this disorder of the nervous system, if such it really is, is no such simple matter as the above, nor have such results as I have been able to obtain been by any means so satisfactory. My first case was that of an intimate friend some years my senior, and although I obtained some credit for my success, I am afraid that I appropriated to my own use the experience of another as I heard him discuss it in lectures, and in less formal fashion. It was from him that I learned of the importance of the increased temperature reading, "whatever the true pathology may be, all that we certainly know of it is that the patient has fever, and presents the symptom of vigillation."

More than once I heard him speak of one of his patients as being a victim, but I never heard him express any opinion in respect to the symptoms completing the picture, nor the cause of the phenomena. In twenty years I have seen some six or eight cases, one case I have seen repeatedly, before, during and after the attacks. In this particular case I have seen this symptom, the vigillation and its companion,(I dare not say consequent, fever; although at times I have suspected the fever to be of nervous origin and have acted accordingly) find its sequel in a short but exceedingly heavy sleep of a few hours' duration; in no visible illness, but extreme weariness and transient weakness and incapacity; in a headache accompanied by an unquestionable delirium of persons, place and circumstance.

The treatment method which has appeared the least useless seeks to obtain relief from the fever by small but repeated, every 15 to 30 minutes, doses of amorphous acontine ½ mg. granule and 5 mgs. of the resenoid of cannabis indica at a dose, followed by 1 mg. (cannabin) every fifteen minutes unless she sleeps.

Why she can tolerate the cannabis indica and not any opium products except codeia is a mystery. Codeine in the form of the sulphate is usually well tolerated and is sometimes substituted for the resinoid in doses of 1-3 grain, if the cannabis indica does not produce immediate effect.

This treatment is effective, produces results, sometimes by, sending the unfortunate patient to sleep, but in this way only. Some dozen men of the widest reputation have seen this patient to no purpose. Occasionally relief is obtained in the way indicated, more frequently the patient goes suddenly to sleep. I have known her to do so leaving a question she was asking unfinished. Such is the typical case of the group which I have denominated cases of vigillation. In the present state of our information the conditions surrounding such a case must be studied as a special problem in pathology and therapeutics, each time the unsteady action of the automatic selfcontrol of nervous reactions manifests itself, and the symptoms met seriatim, because we really are quite without any definite ground on which to build.

THE SINCLAIR HOTEL, 163 FULTON STREET.

